# Knowledge of cancer genetics and attitudes about genetic counseling and testing: a randomized trial of the Know Your Risk intervention compared to conventional genetic counseling

**DOI:** 10.1007/s10552-026-02207-3

**Published:** 2026-06-30

**Authors:** Mira L. Katz, Patrick M. Schnell, Paul L. Reiter, Leigha Senter, Amber Aeilts, Christina Spears, Julia Cooper, Jordan Brown, Kate P. Shane-Carson, Doreen M. Agnese, Amanda E. Toland, Kevin Sweet

**Affiliations:** 1 Division of Health Behavior and Health Promotion, College of Public Health, The Ohio State University, Columbus, OH USA; 2 Comprehensive Cancer Center, The Ohio State University, Columbus, OH USA; 3 Division of Biostatistics, College of Public Health, The Ohio State University, Columbus, OH USA; 4 Division of Human Genetics, Department of Internal Medicine, College of Medicine, Wexner Medical Center, The Ohio State University, Columbus, OH USA; 5 Department of Surgery, College of Medicine, The Ohio State University, Columbus, OH USA; 6 Department of Cancer Biology and Genetics, College of Medicine, Wexner Medical Center, The Ohio State University, Columbus, OH USA; 7 The Ohio State University, Room 212, 3650 Olentangy River Road, Columbus, OH 43214 USA

**Keywords:** Genetic counseling, Genetic testing, Breast cancer, Female

## Abstract

**Purpose:**

To determine if the developed *Know Your Risk* (KYR) intervention is similar to conventional genetic counseling; we evaluated participants’ knowledge of cancer genetics, breast cancer risk perception, attitudes about genetic counseling and testing, and satisfaction with genetic counseling.

**Methods:**

Women (*n* = 866) who screened at elevated risk for breast cancer were randomized to the KYR intervention or conventional genetic counseling (2022–2024). The KYR intervention included a series of online pre-test educational videos, direct access to genetic testing, and patient preference for receiving post-test genetic counseling. Participants completed surveys at baseline and after genetic counseling and testing. Non-inferiority hypothesis testing compared the two participant groups.

**Results:**

The mean knowledge score (range 0–12) increased in both study groups from a baseline mean of 6.87 (standard deviation (SD) = 2.46) to 8.66 (SD = 2.16) for the KYR intervention and 8.56 (SD = 2.14) for conventional counseling. Additionally, statistical analyses suggest the non-inferiority of the KYR intervention for participants’ accuracy of their breast cancer risk, attitudes about genetic counseling and genetic testing, and satisfaction with genetic counseling compared to participants randomized to conventional genetic counseling.

**Conclusion:**

Findings support that the components included in the KYR intervention are non-inferior for cancer genetic knowledge, risk perception, genetic counseling and testing attitudes, and genetic counseling satisfaction among women who screen at elevated risk for breast cancer. By using the combination of components included in the KYR intervention, genetic counseling and genetic testing are more accessible, convenient, and patient-driven.

*Trial Registration*: ClinicalTrials.gov Identifier: NCT05325151.

## Introduction

Approximately 15% of women living in the United States (U.S.) over age 35 are at elevated risk for breast cancer (≥ 20% lifetime) as defined by risk models based on family history [1, 2]. The National Comprehensive Cancer Network (NCCN) recommends that women at elevated risk for breast cancer complete genetic counseling as part of the genetic testing process [3]. This recommendation is important given that genetic counseling and genetic test results may provide information women can use to mitigate their risk by making behavioral changes, including receiving more frequent screening or additional tests (e.g., breast magnetic resonance imaging [MRI]) [3, 4].

Conventional genetic counseling includes a session before and after genetic testing and is completed either in-person, by telephone, or by video conferencing [3]. During the conventional pre-test genetic counseling session, a genetic counselor collects detailed personal and family medical history and assesses the risk of a hereditary cancer predisposition syndrome. In addition, the genetic counselor provides information about genetic testing including possible results, benefits, limitations, and implications for the individual and their family members. During conventional post-genetic test sessions, the genetic counselor helps patients understand their test results (positive, negative, uncertain variant) and provides personalized guideline-indicated recommendations and psychosocial support.

With the upsurge in precision medicine and women undergoing routine breast cancer screening, there has been an increase in the demand for cancer genetic testing while there is a documented shortage of genetic counselors to guide women through the process [4, 5]. To improve patient access and to meet the increasing workforce demand, alternative strategies for genetic counseling, including educational videos, group counseling, decision aids, chatbots, and other interactive technologies have been and continue to be explored [6–9]. These different strategies are proposed to replace or supplement portions of the conventional genetic counseling process and are important to explore given the increased incorporation of genomic information in precision medicine and the acceptance and demand of genetic testing by the public [4, 6].

We developed the online *Know Your Risk* (KYR) intervention for women who screened at elevated risk (> 20% lifetime risk) for breast cancer. In brief, the KYR intervention includes a series of pre-test educational videos, direct access genetic testing, and the opportunity for the patient to drive the content of the post-test genetic counseling session and specify their preference for the communication channel (e.g., video conferencing) to receive post-test genetic counseling. The KYR pre-test educational videos used a narrative arc across the videos and were guided by the Protection Motivation Theory (PMT) [10]. PMT constructs included in the threat and coping appraisals were addressed in the videos (e.g., self-efficacy: character in the video speaking to their friend states “Completing the test was not hard at all. The instructions were easy to follow, and I was able to do everything through the mail.”). A detailed description of the development of the KYR videos has been published [11].

Participants were randomized in a non-inferiority trial to either conventional pre- and post-test genetic counseling or the developed KYR intervention. This report focuses on participants’ cancer genetics knowledge, accuracy of breast cancer risk, attitudes and barriers to genetic counseling and genetic testing, and satisfaction with genetic counseling.

## Materials and methods

### Participants

**Fig. 1:**
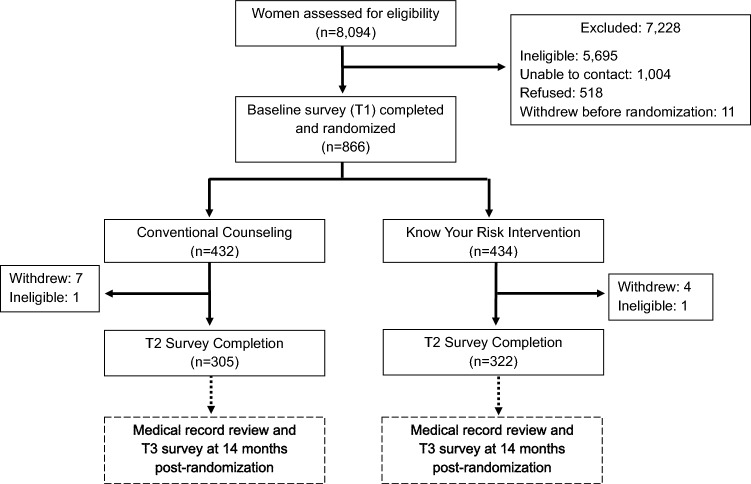
Study flow diagram for participants in the randomized controlled trial

From July 2022 to September 2024, patients (*n* = 8,094) at The Ohio State University Medical Center (OSUMC) who underwent mammography testing and screened at elevated risk for breast cancer were assessed for participation in the current study (Fig. 1). Participants’ breast cancer risk was estimated based on a tool that incorporates a clinically validated cancer risk assessment model (Tyrer-Cuzick versions 7 and 8) [12]. Eligibility for the randomized controlled trial included: (a) being female; (b) ages 30–64; (c) using the OSUMC electronic medical record (EMR) patient portal; (d) undergoing routine screening mammography; (e) having normal Breast Imaging Reporting and Data System (BI-RADS) 1–2; (f) screening at elevated risk for breast cancer by the assessment tool; and (g) ability to read and speak English. The Institutional Review Board at The Ohio State University approved this study.

### Enrollment and randomization

Patients’ medical charts were reviewed, and 5,695 patients were ineligible because they did not meet study eligibility criteria. A message about the study was sent through the EMR patient portal to potentially eligible patients. If there was no response to the message after two business days, a study recruiter attempted to contact the patient by telephone up to three times on different days and times. Recruiters did not reach 1,004 patients and 518 patients declined study participation. For patients who were eligible and interested in the study, the recruiter sent an email with a link to documents [e.g., electronic study consent, Health Insurance Portability and Accountability Act (HIPAA), and medical record release form] hosted on Research Electronic Data Capture (REDCap). As part of the consent process, the study recruiter reviewed the study documents with potential participants and answered any study-related questions.

After signing the study documents, patients were sent an email with a link to the baseline survey (T1) hosted on REDCap. Eleven patients who signed the study consent form withdrew from the study prior to completing the baseline survey. Consented patients who completed the baseline survey (*n* = 866) were sent a reminder to review and update their family history in their EMR and were randomized in a 1:1 allocation to conventional genetic counseling or the KYR intervention.

### Conventional genetic counseling

Participants assigned to conventional genetic counseling were contacted by the clinical genetics department and offered in-person, telephone, or video conferencing for their initial genetic counseling session. If participants completed genetic testing, they were contacted by the genetic counselor when the genetic test results were available for their post-test counseling session. Participants were sent an email with a link to a second survey (T2) if: (a) after three attempts they did not schedule an initial genetic counseling session, (b) they decided against genetic testing after the initial genetic counseling session, (c) after post-test genetic counseling, or (d) after three failed attempts to arrange a post-test genetic counseling session.

### Know your risk intervention

The KYR intervention was developed to improve the genetic counseling and genetic testing experience and adherence to NCCN recommendations for women who screened at elevated breast cancer risk [11]. Participants assigned to the KYR intervention were sent an email with a link to the online study landing page. The landing page included links to access the study introductory video and six educational videos with the following titles: Breast Cancer Information, Genetic Testing and Genetic Counseling, Elevated Risk for Breast Cancer, Know Your Risk, Genetic Test Results, and Talking to Family Members. The educational videos ranged from 2 min 42 s to 6 min 23 s.

Participants were able to view each video at their convenience, and as many times as they wanted during the study. After viewing each video, participants were presented with a pop-up screen where they could prompt a request for genetic testing and/or watch the next video. Participants could also request a genetic test directly from a link on the study’s landing page. In addition, participants could email or call the study recruiter with questions, and a designated genetic counselor would assist the recruiter with the response, if needed. Following genetic testing, participants were able to drive the content and communication channel for their post-test genetic counseling session by completing a survey sent to them in the EMR patient portal.

Participants in the KYR intervention group were sent an email with a link to a second survey (T2) if: (a) they did not request genetic testing, (b) they decided against genetic testing, (c) after post-test genetic counseling, or (d) after three failed attempts to arrange a post-test genetic counseling session.

### Genetic testing

All participants requesting genetic testing were mailed a study-specific saliva sample collection test kit from Myriad Genetics, Inc. Participants who returned a saliva sample to the clinical laboratory improvement amendments (CLIA)-approved laboratory were tested with a 48-gene hereditary cancer panel. The test also evaluated a combined risk score (CRS) that integrated Tyrer-Cuzick version 8.0 (TC8) breast cancer risk modeling with a multiple-ancestry polygenic risk score and a separate TC8 score. CRS and TC8 risk scores were not calculated if a pathogenic variant (mutation) was identified in any of 13 breast cancer-specific genes (*ATM, BARD1, BRCA1, BRCA2, CDH1, CHEK2, NF1, NTLH1 (biallelic), PALB2, PTEN, RAD51C, RAD51D, STK11, TP53*) on the panel. The laboratory sent the genetic test result reports directly to the OSUMC clinical genetics department.

For participants in the conventional counseling study arm, the assigned genetic counselor contacted participants for post-test counseling that included a review of the panel test and CRS results. If the panel test was negative for pathogenic variants in a breast cancer gene, positive for a pathogenic variant in a non-breast cancer-specific gene (e.g., *APC*) or was a variant of uncertain significance (VUS) in any gene, the genetic counselor recalculated a clinical risk estimate using the TC8 model, the participant’s medical and hormonal history, and their family medical history collected in the pre-test genetic counseling session. If the test was positive for a pathogenic variant in a breast cancer gene (e.g., *BRCA1*), the risk estimates for the specific gene were based on published averages (e.g., NCCN) and were provided to the participant.

For participants assigned to the KYR intervention, the genetic test results were uploaded to the participant’s EMR patient portal along with a genetic counseling patient preference survey to arrange post-test counseling. The survey included prompts to ask whether the participant reviewed and understood their genetic test results, to document specific questions or concerns they had about the test results and their breast cancer risk, to indicate if there were additional questions or concerns about their personal or family medical history to discuss with the genetic counselor, and to specify their preferred communication channel for their post-test genetic counseling session. During the post-test counseling session, the genetic counselor addressed the participants submitted survey questions to establish a participant-driven counseling agenda. The counselor reviewed the panel test and CRS results and conveyed their recalculated TC8 breast cancer risk as described for participants in the conventional counseling arm of the study.

If post-test genetic counseling was completed by participants in both study arms, a summary letter with NCCN screening and management recommendations was sent via the EMR system to participants and their healthcare providers. If post-test counseling was not completed for participants in the conventional genetic counseling study arm, a summary letter was sent to the participant and their provider via the EMR system and included clinical management recommendations based on the test results and/or recalculated TC8 clinical risk estimate. If participants in the KYR intervention group did not complete post-test genetic counseling, a recalculated TC8 clinical risk estimate and screening recommendations were excluded since the participants’ clinical information was unverified. In these cases, the summary letter only provided the test results. Since all participants met NCCN criteria upon enrollment in the study, the cost of genetic testing was charged to the patients’ health insurance, with self-pay and financial assistance options available as needed.

### Measures

The primary endpoints for the trial were adherence to NCCN screening and risk reduction recommendations for breast cancer, and separately for other cancers. We report here the intervention’s short-term effects on cancer genetic knowledge, breast cancer risk perception and beliefs, cancer genetic testing and counseling attitudes, barriers, and satisfaction.

### Demographic and health-related characteristics

Participants’ demographic and health-related characteristics were obtained on the baseline survey (T1). Smoking behavior was assessed using two items and participants were categorized as never, former, or current smokers based on their responses. Health literacy was assessed using the Single Item Literacy Screener and participants were categorized as having “adequate” or “limited” health literacy [13]. We assessed numeracy with the eight-item Subjective Numeracy Scale [14] and scores were summed (range 8–48), with higher scores indicating higher subjective numeracy (perceived ability and preference for numeric information).

### Cancer genetic knowledge

To assess cancer genetic knowledge, we used a 12-item scale that was modified from a validated cancer genetic knowledge scale on the T1 and T2 surveys [15]. Response options for each item were “agree,” “disagree,” or “don’t know.” Responses of don’t know were considered incorrect. Correct answers were summed, with higher scores indicating better cancer genetic knowledge (range 0–12).

### Breast cancer risk perception and beliefs

We assessed breast cancer risk perception on the T1 and T2 surveys with one open-ended item: “I think my risk for developing breast cancer in my lifetime is:__,” and the participant wrote in their percent chance (range 0–100). The participants’ perceived risk (percentage) of breast cancer was considered accurate if it was within ± 10 percentage points from the genetic counselor’s calculated TC8 risk [16]. The TC8 risk calculated by the genetic counselor during the post-test genetic counseling session was used when available. If no post-test counseling was completed in the conventional arm, we used the TC8 score computed by the counselor based on the participant’s medical, hormonal, and family history collected in the pre-test counseling session. If no post-test counseling was completed in the KYR intervention arm, we used the Myriad TC8 score available in the test report. If testing revealed a genetic pathogenic or likely pathogenic variant relevant to breast cancer risk, the risk range corresponding to that variant was used, with accurate risk perception defined as up to 10 percentage points below the lower bound and up to 10 percentage points above the upper bound.

In addition, perceived susceptibility of breast cancer was assessed with one item: “The chance that I might develop breast cancer is high” and breast cancer severity was assessed with one item “Breast cancer is a serious disease.” Both items address constructs in the PMT’s threat appraisal and had response options on a 5-point scale (“strongly agree” to “strongly disagree”) with a range of 1–5 for each item.

To address additional key constructs in the PMT’s threat and coping appraisal, the following genetic counseling and genetic testing attitudes and barriers were also measured.

### Cancer Genetic Counseling and Testing Attitudes

The surveys included 9 items to measure attitudes about cancer genetic counseling (e.g., benefit: “Genetic counseling will help me learn about what I can do about my cancer risk”) and 7 items about genetic testing attitudes (e.g., response-efficacy: “genetic tests will give me accurate information”). Responses were on a 5-point Likert scale from “strongly agree” to “strongly disagree.” Items were summed and higher scores reflect more positive attitudes about genetic counseling (range 9–45) and testing (range 7–35).

### Genetic counseling and genetic testing barriers

Genetic counseling and/or genetic testing barriers were measured in the T2 survey among participants who did not complete genetic counseling and/or genetic testing. We used 4 items to measure genetic counseling barriers and 9 items to assess genetic testing barriers with response options on a 5-point Likert scale (“strongly agree” to “strongly disagree”). Items were summed and higher scores reflect more barriers to genetic counseling (range 4–20) and/or more barriers to genetic testing (range 9–45). Next, a list of 13 common barriers (e.g., cost) was provided for genetic counseling and for genetic testing on the survey and participants could indicate multiple barriers from each list. Finally, participants responded to items focused on intention to complete genetic counseling or genetic testing in future on a 5-point Likert scale with higher scores indicating more intention to complete genetic counseling or testing in future (range 1–5).

### Genetic counseling satisfaction

Among participants who completed genetic counseling, satisfaction with genetic counseling scale was included in the T2 survey. The scale included 10 items with response options on a 5-point Likert scale (“strongly agree” to “strongly disagree”). Six items were from a validated scale [17] and 4 additional items were developed by the investigators. The range of scores was 6–30 if the 6 items were evaluated and 10–50 for the 10 items. Higher scores indicate greater satisfaction with genetic counseling. In addition, we included one summary item “On a scale of 1 to 10, with 1 being dissatisfied and 10 being satisfied, how would you rate your satisfaction with your genetic counseling session?”.

### Data analysis

All data analyses were conducted using R version 4.5.0. Analyses of changes from baseline used the intention-to-treat strategy, grouping all randomized participants by assignment to the two study arms: the KYR intervention group and the conventional genetic counseling group. Analyses of counseling satisfaction were restricted to subjects who took part in counseling. We computed descriptive statistics for baseline demographic and health-related characteristics of participants.

Baseline knowledge score was assumed to be unchanged for subjects who opted to participate in none of the components of either arm. To evaluate non-inferiority of the KYR intervention versus conventional counseling, we used a non-inferiority margin of 0.6 on the odds ratio scale and used mixed-effects logistic regression to estimate the intervention effect adjusted for baseline variables to improve precision. Random intercepts were included for genetic counselors, and fixed effects were included for study arm, baseline breast cancer knowledge, and baseline perception of breast cancer risk. Simple proportions were also computed as unadjusted summaries for each arm. Analysis of risk perception accuracy proceeded similarly to that for genetic knowledge. Mixed-effects logistic regression accounts for missing outcome data under the assumption of missingness-at-random conditional on the fixed and random effect variables.

Participants’ attitudes about genetic counseling and genetic testing were summarized by mean and standard deviation (SD), and a two-sample t-test was used to compare change from baseline for testing attitudes among those who were tested between arms. Frequencies were calculated for barriers to genetic counseling and genetic testing reported by participants.

The primary satisfaction measure was the 6-item scale. We evaluated non-inferiority of KYR using a non-inferiority margin of −1 point and used mixed-effects linear regression to estimate the intervention effect, with the same random and fixed effects as for genetic knowledge. Similar models were also fit to the 10-item scale and the single-item. As a sensitivity analysis, a composite endpoint was used that considered patients who either declined to complete a genetic test or tested but did not choose to participate in post-test genetic counseling to hold the least favorable satisfaction rating.

### Power and sample size

We aimed to randomize 1000 subjects in total, expecting complete data on approximately 700 (350 per arm). Assuming equivalence of study arms, this enrollment target was estimated to yield at least 80% power to conclude non-inferiority with a margin of 0.6 on the odds ratio scale for the binary endpoints of NCCN breast cancer recommendation adherence (primary), other cancer screening recommendation adherence, breast cancer genetic knowledge, and accuracy of risk perception. Over 94% power to conclude non-inferiority was estimated for genetic counseling satisfaction with a margin of 1 point on the 6-to-30 scale. Detailed study information is available in the published protocol [18]. As no single endpoint alone would be sufficient to establish non-inferiority overall, no adjustment for multiple testing was used.

## Results

### Participant characteristics

The average age of participants was 47.8 years (Table 1). Most participants were non-Hispanic (95.5%), White (83.1%), were married or living with a partner (74.2%), had at least a college degree (79.0%), and were employed full time (77.5%). About half of the participants reported annual household incomes greater than $120,000 (45.4%), and that religion was fairly, very, or extremely important to them (50.6%). Participants’ political view was conservative/somewhat conservative (16.0%), moderate (20.7%), or somewhat liberal/liberal (50.0%). Only 5.9% of participants were current smokers and 1.7% had limited health literacy. Most participants (99.3%) had some form of health insurance.

**Table 1:** Demographic characteristics of participants (*n* = 866)

	Conventional (*n* = 432)	KYR Intervention (*n* = 434)
Age, years [mean (SD)]	48.0 (7.65)	47.7 (7.49)
	N (%)	N (%)
Ethnicity		
Hispanic	19 (4.4)	14 (3.2)
Non-Hispanic	411 (95.1)	416 (95.9)
Missing	2 (0.5)	4 (0.9)
Race		
White	356 (82.4)	364 (83.9)
Black	56 (13.0)	49 (11.3)
Asian	4 (0.9)	5 (1.2)
American Indian/Alaska Native	1 (0.2)	1 (0.2)
Two or more races	10 (2.3)	10 (2.3)
Another race not listed	4 (0.9)	3 (0.7)
Missing	1 (0.2)	2 (0.5)
Marital status		
Never married	56 (13.0)	43 (9.9)
Married or living with partner	317 (73.4)	326 (75.1)
Separated or divorced	48 (11.1)	59 (13.6)
Widowed	7 (1.6)	4 (0.9)
Missing	4 (0.9)	2 (0.5)
Education level		
Less than high school	1 (0.2)	1 (0.2)
High school/GED	29 (6.7)	38 (8.8)
Some college/Technical School	51 (11.8)	61 (14.1)
College degree	147 (34.0)	152 (35.0)
Graduate/Professional	204 (47.2)	181 (41.7)
Missing	0	1 (0.2)
Employment		
Full time	345 (79.9)	326 (75.1)
Part time	27 (6.3)	41 (9.4)
Stay at home	22 (5.1)	17 (3.9)
Not employed	9 (2.1)	18 (4.1)
Retired	16 (3.7)	20 (4.6)
Disabled	11 (2.5)	8 (1.8)
Missing	2 (0.5)	4 (0,9)
Household income		
Less than $20,000	17 (3.9)	10 (2.3)
$20,000 to $39,999	13 (3.0)	24 (5.5)
$40,000 to $69,999	45 (10.4)	50 (11.5)
$70,000 to $99,999	66 (15.3)	50 (12.7)
$100,000 to $119,999	52 (12.0)	64 (14.7)
$120,000 +	201 (46.5)	192 (44.2)
Do not know	4 (0.9)	2 (0.5)
Missing	34 (7.9)	37 (8.5)
Political view		
Very Conservative	19 (4.4)	14 (3.2)
Somewhat conservative	53 (12.3)	53 (12.2)
Moderate	86 (19.9)	93 (21.4)
Somewhat liberal	108 (25.0)	125 (28.8)
Very Liberal	111 (25.7)	89 (20.5)
Missing	55 (12.7)	60 (13.8)
Religiosity		
Not at all or slightly important	197 (45.6)	187 (43.1)
Fairly, very, or extremely important	214 (49.6)	225 (51.8)
Missing	21 (4.9)	22 (5.1)
Sexual identity		
Straight or heterosexual	395 (91.4)	405 (93.3)
Some other identity	31 (7.1)	22 (5.1)
Health-Related Characteristics		
Health insurance		
Yes	431 (99.8)	429 (98.8)
No	0	4 (0.9)
Missing	1 (0.2)	1 (0.2)
Smoking status		
Current	23 (5.3)	28 (6.5)
Former	98 (22.7)	88 (20.3)
Never	300 (69.4)	303 (69.8)
Missing	11 (2.5)	15 (3.5)
Health literacy		
Limited	8 (1.9)	7 (1.6)
Adequate	424 (98.1)	427 (98.4)
Subjective Numeracy mean (SD)	38.9 (6.9)	38.9 (7.0)

Subjective Numeracy: scores range from 8 to 48, higher scores indicate higher subjective numeracy

*SD* standard deviation

### Genetic testing, post-test genetic counseling, and T2 survey completion

Genetic testing was completed by 77.6% (672/866) of participants including 81.8% (355/434) of participants randomized to the KYR intervention and 73.4% (317/432) of participants randomized to conventional genetic counseling. Post-test genetic counseling was completed by 91.3% (324/355) of participants in the KYR intervention group and by 98.4% (312/317) of participants randomized to conventional genetic counseling. The second survey (T2) was completed by 322 (74.2%) participants in the KYR intervention group and by 305 (70.6%) participants in the conventional counseling group.

### Cancer genetic knowledge

The genetic knowledge mean (SD) score (Table 2) at baseline was 6.87 (SD = 2.46). The mean score increased in both study arms on the T2 survey; conventional genetic counseling mean score was 8.56 (SD = 2.14) and KYR intervention mean score was 8.66 (SD = 2.16). Examples of items that increased in both groups were: “The lifetime chance of getting cancer depends on which altered cancer gene is inherited,” “A Variant of Uncertain Significance (VUS) will not likely influence recommendations for screening or prevention,” and “All children of a person with inherited cancer risk will also have inherited cancer risk.” The unadjusted proportions of subjects with post-intervention knowledge scores ≥ 9 were comparable between participants randomized to the KYR intervention (58.8%) and those assigned to conventional genetic counseling (58.6%) (adjusted odds ratio (OR) 1.36; 95% confidence interval (CI) 0.92 to 2.00; *p* = 0.12). The one-sided 95% lower confidence bound for the effect of KYR intervention vs. conventional genetic counseling is 0.98 on the odds ratio scale, within the non-inferiority margin of 0.6. Thus, there is statistical evidence that the KYR intervention is non-inferior to the conventional genetic counseling approach within an OR margin of 0.6.

**Table 2:** Genetic knowledge among participants at baseline (T1) and on the T2 surveys

Items	Correct answer	Baseline T1 survey (*n* = 866) n (%)	Conventional counseling T2 survey (*n* = 324) n (%)	KYR intervention T2 survey (*n* = 354) n (%)
Knowing about inherited risk (passed down within a family) can affect choices about cancer prevention (for example, surgery)	Agree	830 (95.8)	317 (97.8)	350 (98.9)
People with an inherited risk for cancer (and their at-risk family members) are more likely to develop more than one type of cancer	Agree	457 (52.8)	198 (61.1)	211 (59.6)
A person with inherited risk for cancer will definitely get cancer one day	Disagree	637 (73.6)	280 (86.4)	316 (89.3)
The lifetime chance of getting cancer depends on which altered cancer gene is inherited	Agree	411 (47.5)	227 (70.1)	253 (71.5)
People with a higher than average risk for cancer may get cancer at a younger age than people with average risk	Agree	448 (51.7)	224 (69.1)	235 (66.4)
In future, more information could become available that could alter the meaning of genetic test results	Agree	759 (87.6)	306 (94.4)	333 (94.1)
All children of a person with inherited cancer risk will also have inherited cancer risk	Disagree	438 (50.6)	236 (72.8)	260 (73.4)
Family members (for example, sister, father, or child) of a person with a harmful variant in a cancer gene might share the same gene variant	Agree	733 (84.6)	297 (91.7)	329 (92.9)
Female-specific cancer risk, such as ovarian cancer, can generally be passed on from either the father or mother	Agree	320 (37.0)	176 (54.3)	194 (54.8)
In most cases, the children of a person with inherited cancer risk have a 50–50 (50%) chance of having inherited risk for cancer too	Agree	320 (37.0)	172 (53.1)	191 (54.0)
Some harmful gene variants mean a larger increase in the risk for cancer while others mean a smaller increase in the risk for cancer	Agree	456 (52.7)	209 (64.5)	247 (69.8)
A Variant of Uncertain Significance (VUS) will not likely influence recommendations for screening or prevention	Agree	142 (16.4)	132 (40.7)	145 (41.0)

*KYR* Know Your Risk

### Breast cancer risk perception and beliefs

The unadjusted proportions of subjects with post-intervention risk perception accurate to within 10 percentage points were higher in the KYR intervention group (88.4%) compared to the conventional genetic counseling group (78.8%) (adjusted OR 1.98; 95% CI 1.21 to 3.27, *p* = 0.01). The one-sided 95% lower confidence bound for the effect of KYR intervention vs. conventional genetic counseling is 1.31 on the odds ratio scale, within the non-inferiority margin of 0.6. Thus, there is statistical evidence that the KYR intervention is non-inferior to conventional genetic counseling with an OR margin of 0.6.

At baseline, 96% of participants strongly agreed that breast cancer is a serious disease, and 97% at the T2 survey. However, for the statement “The chance that I might develop breast cancer is high,” participants in both groups at baseline had a median score (39% of responses) of slightly agree with that statement and participants in both groups changed their response to a median score of slightly disagree (34% in conventional genetic counseling, 39% in KYR intervention).

### Cancer genetic counseling and testing attitudes

Participant attitudes about genetic counseling at baseline were positive with a mean score (SD) for conventional genetic counseling 36.9 (SD = 4.3) and for the KYR intervention 37.2 (SD = 4.5) on a total scale range of 9–45. Genetic testing attitudes on a 10–50 scale were also slightly positive at baseline: 33.6 (SD = 3.5) for participants in the conventional genetic counseling group and 33.7 (SD = 3.5) for KYR participants. After testing, mean testing attitude score was higher in the KYR intervention arm than in the conventional genetic counseling arm (35.1 vs 34.5, 95% CI for difference in change from baseline 0.1 to 1.1, *p* = 0.03). Because only those who were tested reported post-testing attitudes, mean changes from baseline do not represent causal effects, including comparisons between arms.

### Genetic counseling and genetic testing barriers

Among the 20 participants who completed the items focused on barriers to genetic counseling and genetic testing, 14 were in the KYR intervention group and 6 were in the conventional genetic counseling group. The two most frequent barriers reported by participants in both arms of the study were cost (40%, 8/20) and no time (40%, 8/20) for genetic counseling and cost (35%, 7/20) and no time (45%, 9/20) for genetic testing. All other barriers were reported by fewer than 5 of the 20 participants.

### Genetic counseling satisfaction

For the 6 items from the validated scale (range 6–30), the unadjusted mean satisfaction score was comparable between the KYR intervention group (28.5) and the conventional genetic counseling group (28.5) (adjusted difference − 0.01; 95% CI − 0.48 to 0.48, *p* = 0.97). The one-sided 95% lower confidence bound for the effect of KYR intervention vs. conventional genetic counseling is − 0.4 on the difference scale, within the non-inferiority margin of − 1. Thus, there is statistical evidence that the KYR intervention is non-inferior to conventional genetic counseling with a score difference margin of − 1.

For the 10 items (range 10–50), the unadjusted mean satisfaction score was comparable between the KYR intervention group (47.3) and conventional genetic counseling group (46.6) (unadjusted difference 0.6; 95% CI − 0.04 to 1.25; *p* = 0.07). There is statistical evidence that the KYR intervention is non-inferior to conventional genetic counseling with the 10 items.

In addition, for the one summary item about genetic counseling satisfaction (range 1–10), the unadjusted mean was slightly higher in the KYR intervention group (9.7) compared to a mean of 9.5 in the conventional genetic counseling arm (adjusted difference 0.2; 95% CI 0.03 to 0.37, *p* = 0.02).

For all satisfaction endpoints, the composite endpoint sensitivity analyses counting subjects who either declined to complete a genetic test or tested but did not choose to participate in post-test genetic counseling as holding the least favorable satisfaction also resulted in conclusions of non-inferiority.

## Discussion

The KYR intervention was developed as an alternative strategy for genetic counseling and testing among women who screened at elevated risk for breast cancer and was modeled after direct-to-consumer genomic-based approaches that integrate web-based knowledge aids and preferences into the user experience for increased access, personalization, and convenience [19, 20]. The KYR intervention included a series of online pre-test educational videos, direct access to genetic testing, and patient preference for receiving post-test genetic counseling that incorporates a patient-driven agenda. The KYR videos used a narrative format, were guided by the PMT, and theoretical constructs were measured in the surveys. The KYR intervention was also developed with the inclusion of NCCN guidelines when developing eligibility criteria, using an appropriate CLIA-approved multigene panel test, and NCCN guideline-based management strategies, while increasing access and encouraging active patient engagement.

This report evaluated genetic knowledge, accuracy of breast cancer risk, attitudes about genetic counseling and testing, and satisfaction with genetic counseling among participants in the randomized controlled trial evaluating the KYR intervention. Results show that the KYR intervention is non-inferior to conventional genetic counseling for participants’ cancer genetic knowledge, accuracy of breast cancer risk, having positive attitudes about genetic counseling and genetic testing, and satisfaction with genetic counseling [21–23]. In addition, participants in both study arms showed a high uptake of genetic testing compared to other studies [7].

Although many women have reported that they want information about their personal risk of breast cancer [24, 25], most women remain uncertain of their actual risk and tend to overestimate or underestimate their risk [25]. The genetic counseling process addresses this issue by including the accurate assessment of clinically derived risk and the numeric probability of breast cancer. During the genetic counseling session, the genetic counselor reviews the objective breast cancer risk as well as appropriate strategies to manage this risk with patients. In our study, participants were counseled as to the meaning of the CRS included in the genetic test report and how this risk level was derived. The genetic counselor then contrasted the CRS with the recalculated TC8 score, with the latter score utilized to determine specific recommendations for breast cancer screening and management. Our results support that the KYR intervention is non-inferior to conventional genetic counseling to help women who screened at elevated risk for breast cancer to understand their breast cancer risk and the meaning of their genetic information. Although CRS is not currently part of models used in genetic counseling, it potentially may become a factor for screening decisions for cancer, heart disease, and other conditions in future [26].

The KYR intervention was developed to address the reported important components of pre-test genetic counseling (e.g., risk factors for breast cancer; genetic testing benefits and limitations) while using EMR patient portals to make genetic testing and post-test counseling more accessible and participatory. The emergence of direct access genetic counseling service delivery models is ongoing, driven by advances in technology that lower the cost and provide greater access to genetic/genome data, often facilitated through patient user interfaces (e.g., EMR patient portals) allowing for the efficient provision of clinical information and resources at the point of care.

The strengths of the study include a large sample of women who screened at elevated risk for breast cancer, a randomized controlled trial design, and measuring key factors to determine non-inferiority of the KYR intervention and conventional genetic counseling. Study limitations include that the study was conducted in one health system located in the Midwest U.S., the intervention was web-based, and the findings do not represent all women who screen at elevated risk for breast cancer because the majority of participants were college-educated, non-Hispanic Whites, and reported annual household incomes ≥ $100,000. In addition, only women who met NCCN criteria for genetic testing were eligible to participate in the study, and not all participants completed the T2 survey.

The study results are favorable for the KYR intervention among women who screen at increased risk for breast cancer. Given that the intervention is non-inferior to conventional genetic counseling for cancer genetic knowledge, attitudes about genetic counseling and genetic testing, and satisfaction with genetic counseling, the intervention can be implemented to encourage patient engagement, preference, and convenience. Our future efforts will determine the primary outcomes of the study which focus on participants following the genetic counselors’ recommendations for cancer screening(s) and other health-related behavioral modifications (e.g., smoking cessation).

## Data Availability

The datasets analyzed during the current study are not publicly available at this time but are available from the corresponding author on reasonable request.

## References

[1] Freedman AN, Graubard BI, Rao SR, McCaskill-Stevens W, Ballard-Barbash R, Gail MH (2003) Estimates of the number of US women who could benefit from tamoxifen for breast cancer chemoprevention. J Natl Cancer Inst 95(7):526–532. 10.1093/jnci/95.7.526 12671020 10.1093/jnci/95.7.526

[2] Sabatino SA, Burns RB, Davis RB, Phillips RS, Chen Y, McCarthy EP (2004) Breast carcinoma screening and risk perception among women at increased risk for breast carcinoma: Results from a national survey. Cancer 100(11):2338–2346. 10.1002/cncr.20274 15160336 10.1002/cncr.20274

[3] National Comprehensive Cancer Network (2025 March) Genetic/familial high-risk assessment: Breast, ovarian, pancreatic, and prostate. National Comprehensive Cancer Network; https://www.nccn.org/guidelines/guidelines-detail?category=2&id=1545 10.6004/jnccn.2026.0007 41671423

[4] Collins FS, Varmus H (2015) A new initiative on precision medicine. N Engl J Med 372(9):793–795. 10.1056/NEJMp1500523 25635347 10.1056/NEJMp1500523 PMC5101938

[5] Hoskovec JM, Bennett RL, Carey ME, DaVanzo JE, Dougherty M, Hahn SE et al (2018) Projecting the supply and demand for certified genetic counselors: a workforce study. J Genet Couns 27(1):16–20. 10.1007/s10897-017-0158-8 29052810 10.1007/s10897-017-0158-8

[6] Buchanan AH, Rahm AK, Williams JL (2016) Alternate service delivery models in cancer genetic counseling: a mini-review. Front Oncol 6:120. 10.3389/fonc.2016.00120 27242960 10.3389/fonc.2016.00120 PMC4865495

[7] Kaphingst KA, Kohlmann WK, Lorenz Chambers R, Bather JR, Goodman MS, Bradshaw RL et al (2024) Uptake of cancer genetic services for chatbot vs standard-of-care delivery models: the BRIDGE randomized clinical trial. JAMA Netw Open 7(9):e2432143. 10.1001/jamanetworkopen.2024.32143 39250153 10.1001/jamanetworkopen.2024.32143 PMC11385050

[8] Sie AS, Spruijt L, van Zelst-Stams WAG, Mensenkamp AR, Ligtenberg MJL, Brunner HG et al (2016) High satisfaction and low distress in breast cancer patients one year after BRCA-mutation testing without prior face-to-face genetic counseling. J Genet Couns 25(3):504–514. 10.1007/s10897-015-9899-4 26531312 10.1007/s10897-015-9899-4 PMC4868858

[9] Tabor HK, Jamal SM, Yu J, Crouch JM, Shankar AG, Dent KM et al (2017) My46: A web-based tool for self-guided management of genomic test results in research and clinical settings. Genet Med 19(4):467–475. 10.1038/gim.2016.133 27632689 10.1038/gim.2016.133 PMC5352554

[10] Prentice-Dunn S, Rogers RW (1986) Protection motivation theory and preventive health: beyond the health belief model. Health Educ Res 1:153–161. 10.1093/her/1.3.153

[11] Katz ML, Senter L, Reiter PL, Emerson B, Ennis AC, Shane-Carson KP et al (2023) Development of a web-based, theory-guided narrative intervention for women at elevated risk for breast cancer. Patient Educ Couns 106:163–169. 10.1016/j.pec.2022.10.348 36333195 10.1016/j.pec.2022.10.348 PMC10395484

[12] Tyrer J, Duffy SW, Cuzick J (2004) A breast cancer prediction model incorporating familial and personal risk factors. Stat Med 23(7):1111–1130. 10.1002/sim.1668 15057881 10.1002/sim.1668

[13] Morris NS, MacLean CD, Chew LD, Littenberg B (2006) The single item literacy screener: evaluation of a brief instrument to identify limited reading ability. BMC Fam Pract 7:21. 10.1186/1471-2296-7-21 16563164 10.1186/1471-2296-7-21 PMC1435902

[14] Fagerlin A, Zikmund-Fisher BJ, Ubel PA, Jankovic A, Derry HA, Smith DM (2007) Measuring numeracy without a math test: development of the subjective numeracy scale. Med Decis Making 27(5):672–680. 10.1177/0272989X07304449 17641137 10.1177/0272989X07304449

[15] Underhill-Blazey M, Stopfer J, Chittenden A, Nayak MM, Lansang K, Lederman R et al (2019) Development and testing of the KnowGene scale to assess general cancer genetic knowledge related to multigene panel testing. Patient Educ Couns 102(8):1558–1564. 10.1016/j.pec.2019.04.014 31010603 10.1016/j.pec.2019.04.014

[16] Lerman C, Lustbader E, Rimer B, Daly M, Miller S, Sands C et al (1995) Effects of individualized breast cancer risk counseling: a randomized trial. J Natl Cancer Inst 87(4):286–292. 10.1093/jnci/87.4.286 7707420 10.1093/jnci/87.4.286

[17] DeMarco TA, Peshkin BN, Mars BD, Tercyak KP (2004) Patient satisfaction with cancer genetic counseling: a psychometric analysis of the genetic counseling satisfaction scale. J Genet Couns 13(4):293–304. 10.1023/b:jogc.0000035523.96133.bc 19736695 10.1023/b:jogc.0000035523.96133.bc PMC3551590

[18] Sweet K, Reiter PL, Schnell PM, Senter L, Shane-Carson KP, Aeilts A, Cooper J, Spears C, Brown J, Toland AE, Agnese DM, Katz ML (2023) Genetic counseling and testing for females at elevated risk for breast cancer: protocol for the randomized controlled trial of the *Know Your Risk* intervention. Contemp Clin Trials 133:107323. 10.1016/j.cct.2023.107323 37661005 10.1016/j.cct.2023.107323 PMC10591709

[19] Schmidlen T, Sturm AC, Hovick S, Scheinfeldt L, Scott Roberts J, Morr L et al (2018) Operationalizing the reciprocal engagement model of genetic counseling practice: a framework for the scalable delivery of genomic counseling and testing. J Genet Couns 27(5):1111–1129. 10.1007/s10897-018-0230-z 29460110 10.1007/s10897-018-0230-z PMC6098987

[20] Jiang S, Liberti L, Lebo D (2023) Direct-to-consumer genetic testing: a comprehensive review. Ther Innov Regul Sci 57(6):1190–1198. 10.1007/s43441-023-00567-5 37589855 10.1007/s43441-023-00567-5

[21] Culver JO, Bertsch NL, Kurz RN, Cheng LL, Pritzlaff M, Rao SK et al (2024) Systematic evidence review and meta-analysis of outcomes associated with cancer genetic counseling. Genet Med 26(1):100980. 10.1016/j.gim.2023.100980 37688462 10.1016/j.gim.2023.100980 PMC11981685

[22] Matloff ET, Moyer A, Shannon KM, Niendorf KB, Col NF (2006) Healthy women with a family history of breast cancer: impact of a tailored genetic counseling intervention on risk perception, knowledge, and menopausal therapy decision making. J Womens Health (Larchmt) 15(7):843–856. 10.1089/jwh.2006.15.843 16999640 10.1089/jwh.2006.15.843

[23] Smerecnik CMR, Mesters I, Verweij E, de Vries NK, de Vries H (2009) A systematic review of the impact of genetic counseling on risk perception accuracy. J Genet Couns 18(3):217–228. 10.1007/s10897-008-9210-z 19291376 10.1007/s10897-008-9210-z PMC7451018

[24] Fisher BA, Wilkinson L, Valencia A (2016) Women’s interest in a personal breast cancer risk assessment and lifestyle advice at NHS mammography screening. J Public Health (Oxf) 39(1):113–121. 10.1093/pubmed/fdv211 10.1093/pubmed/fdv211 PMC5356472 26834190

[25] Printz C (2014) Most women have an inaccurate perception of their breast cancer risk. Cancer 120(3):314–315. 10.1002/cncr.28557 24452671 10.1002/cncr.28557

[26] Kullo IJ (2025) Clinical use of polygenic risk scores: current status, barriers and future directions. Nat Rev Genet. 10.1038/s41576-025-00900-8 41073616 10.1038/s41576-025-00900-8

